# Schwann Cell-Like Cells Derived from Human Amniotic Mesenchymal Stem Cells Promote Peripheral Nerve Regeneration through a MicroRNA-214/c-Jun Pathway

**DOI:** 10.1155/2019/2490761

**Published:** 2019-07-01

**Authors:** Wei Chen, Shune Xiao, Zairong Wei, Chengliang Deng, Kaiyu Nie, Dali Wang

**Affiliations:** Department of Plastic Surgery, Affiliated Hospital of Zunyi Medical University, Zunyi, Guizhou, China

## Abstract

**Background:**

The use of Schwann cell-like cells (SCLCs) derived from stem cells has been introduced as an effective strategy for promoting peripheral nerve regeneration (PNR). However, molecular mechanisms underlying therapeutic transplantation of SCLCs for PNR are often ignored.

**Objectives:**

To explore the potential of SCLCs for the treatment of sciatic never injury and investigate the underlying molecule mechanisms.

**Method:**

SCLCs differentiated from human amniotic mesenchymal stem cells (hAMSCs) and specific markers of Schwann cells were detected. SCLCs were transplanted into the injured sites of a rat model of sciatic nerve injury, and sciatic nerve functional index (SFI) was determined.

**Results:**

SCLCs expressed specific markers of Schwann cells as well as secreted neurotrophic factors. The transplantation of SCLCs into injured sites of a rat model of sciatic nerve injury promoted the functional recovery. With regard to the underlying molecular mechanisms, we identified c-Jun as a negative regulator of the myelination of SCLCs. Moreover, we discovered a novel signaling transduction pathway in SCLCs; that is, miR-214 directly targets c-Jun to promote the myelination of SCLCs. Finally, we demonstrated that miR-214 upon overexpression in SCLCs enhanced the therapeutic effects of SCLCs on sciatic nerve injury.

**Conclusions:**

We demonstrate that SCLCs have beneficial effect for myelination. Moreover, our results provide a previously unknown molecular basis underlying the treatment of peripheral nerve injury with SCLCs and also offer a practical strategy for future therapeutic promotion of PNR.

## 1. Introduction

The peripheral nervous system (PNS) and the central nervous system (CNS) are the two components of nervous systems. PNS comprises nerves and ganglia outside the brain and spinal cord, and it serves as a link to connect the brain with the rest of the body [1]. Unlike CNS that is protected by the blood-brain barrier, PNS is exposed to toxins and mechanical damage, causing easy injury. In clinics, peripheral nerve injury (PNI) is a commonly encountered problem worldwide, which is mainly caused by traumatic injuries, diabetes, cancer, and surgery [2, 3]. For the treatment of PNI with no loss of nerve tissue or with a short nerve gap, end-to-end suturing is commonly used [4]. However, in most cases, the injury gap between proximal and distal stumps is large and the end-to-end suturing is thus not applicable. Currently, the standard clinical treatment of PNI with a large injury gap is the autologous nerve grafting [5, 6]. Nonetheless, autograft implantation is limited by the availability of donor tissues, donor site morbidity, complications from second surgery, etc. [6, 7]. Therefore, alternative strategies for the repair of PNI are under extensive exploration.

Different from CNS, PNS has a certain capacity for axonal regeneration [8]. During this process, Schwann cells (SCs), the major glia in PNS, proliferate and migrate to the injury site, followed by the formation of Bunger's bands and the secretion of neurotrophic factors to promote peripheral nerve regeneration (PNR) [9, 10]. However, the spontaneous self-regeneration is usually incomplete and may lead to poor repair results. Introducing cultured SCs into the injured sites has demonstrated great potential in animal models to promote axonal regeneration and functional recovery [2, 7, 11]. Nevertheless, the clinical translation of SCs is still limited by the poor feasibility of autologous SCs, sacrifice of functional nerves during isolation, difficulty to culture and expand SCs *in vitro*, and immunologic rejection, as well as the poor proliferative rate and survival of SCs after transplantation into the injured sites [12, 13].

To address these issues, stem cells including human mesenchymal stem cells derived from bone marrow [14, 15], adipose [16, 17], amnion [18], and other tissues [19–21], as well as induced pluripotent stem cells [22–24] have been exploited as alternative cell sources to support PNR. Upon stimulation with appropriate agents *in vitro*, these stem cells differentiate into neuron-like cells or Schwann cell-like cells (SCLCs) [11]. After transplantation into injured sites, these derived cells are able to promote PNR and functional recovery. However, there are still two major obstacles for the clinical use of stem cells in PNR. One obstacle is the unsatisfactory therapeutic outcomes by using current stem cell-based strategies. Although the combined use of stem cells with SCs or other stimuli could further improve PNR [25–27], a more effective strategy is still highly in demand. Another obstacle is the lack of molecular mechanisms underlying the stem cell-based therapy, which raises a concern about the safety issue [28]. Moreover, understanding the molecular mechanisms will also provide a way to enhance the therapeutic effects of stem cells on PNR.

MicroRNAs (miRNAs) are a class of naturally occurring small noncoding RNAs, which consist of ~22 nucleotides and negatively regulate gene expression at the posttranscriptional level [29]. The widespread regulation of miRNAs on the expression of target genes leads to their extensive involvement in both physiologies and pathologies [30, 31]. It is no wonder that miRNAs also play important roles in PNI [32]. For instance, miR-182 inhibits the proliferation and migration of SCs by targeting fibroblast growth factor 9 (FGF9) and neurotrimin (NTM) after sciatic nerve injury [33] and miR-9 inhibits SC migration by targeting collagen triple helix repeat containing protein 1 (CTHRC1) after sciatic nerve injury [34]. It is noteworthy that inhibition of miR-146b is shown to promote PNR following sciatic nerve injury [35]. However, whether miRNAs participate in the stem cell-promoted PNR is still unknown, and whether these miRNAs can be used as targets to enhance the therapeutic outcomes of stem cells on PNR also remains underexplored.

Here we report the use of SCLCs derived from human amniotic mesenchymal stem cells (hAMSCs) for PNR and functional recovery. hAMSCs were differentiated into SCLCs using SC induction medium, and SCLCs were confirmed by analysis of SC markers. We validated the potential of SCLCs in PNR by transplanting them into a rat model of sciatic nerve injury. Moreover, we investigated the molecular mechanism underlying SCLC-promoted PNR, revealing an unknown pathway that is that miR-214 targets c-Jun to promote PNR. We also demonstrated that overexpression of miR-214 in SCLCs could further promote PNR and functional recovery.

## 2. Materials and Methods

### 2.1. hAMSC Extraction and Culture

We chose puerpera under 30 years old to strip the amniotic membrane under aseptic condition. The amniotic membrane was rinsed with PBS to remove residual blood, the amnion was shred and placed in a centrifuge. 0.5 mg/mL type II collagenase and 0.05 mg/mL Dnase I were added and digested for 1 hour, the cell filtrate was collected, and the cells were suspended in DMEM/F12 complete medium containing 10% FBS, 1% L-glutamine, 1% bi-antibody, and 10 ng/mL bFGF; then the medium was incubated in an incubator at 37°C and 5% CO_2_. Cell medium should change after 48 h.

### 2.2. hAMSCs Differentiate into SCLCs In Vitro

The primary hAMSC cells were planted in six hole plates, and differentiation was taken when cell density reached 80-90%. DMEM/F12 differentiation medium contained 1 mmol/L *β*-mercaptoethanol and was incubated at 37°C and 5% CO_2_. The cells were cultured in differentiation medium DMEM/F12 with 1 mmol/L *β*-mercaptoethanol at 37°C and 5% CO_2_. After 24 hours, the medium was changed into DMEM/F12 with 35 ng/mL ATRA and 10% FBS. After 72 hours, the medium was further changed into DMEM/F12 with 10% FBS, 5 mmol/L Forskolin, l0 ng/mL bFGF, 5 ng/mL PDGF-AA, and 200 ng/mL HRG. Thereafter, the medium was refreshed every 3 days.

### 2.3. ELISA

In the process of inducing differentiation of hAMSCs into SCLC in vitro, we chose to detect the amount of related protein expression on days 0, 7, 13, and 19. The cell supernatant was taken, and the expression levels of S100*β*, GFAP, and p75 were detected by the ELISA kit. The absorbance of each sample was measured by a microplate reader, and the content of the target protein in one sample was calculated.

### 2.4. Immunofluorescence

Immunofluorescence staining of hAMSCs and SCLCs that were differentiated from hAMSCs was performed by using the S100*β*, GFAP, or p75 antibody. Briefly, the hAMSC and SCLC cells were first cultured in 24-well plates with glass coverslips for 24 h and further fixed with paraformaldehyde. The cells were then treated with 0.5% Triton X-100; stained with S100*β*, GFAP, and p75 primary antibody; and further stained with FITC-conjugated secondary antibody. After washing with PBS for three times, fluorescence images were then directly taken under a confocal microscope.

### 2.5. Western Blotting

The hAMSC and SCLC cells were washed twice with ice-cold PBS and centrifuged at 12,000 g for 10 min at 4°C, lysed using RIPA lysis buffer containing the protease inhibitor cocktail (Synthgene, China), and incubated on ice for about 30 min. Cell lysates were centrifuged for another 10 min at 4°C (12,000 g). Subsequently, the protein concentration of the supernatant was determined with a BCA protein assay kit (Synthgene, China). The protein levels were then incubated with the following primary antibodies at 4°C overnight: S100*β*, GFAP, p75, c-Jun, Egr2, MBP, MPZ, and GAPDH (Abcam, USA). GAPDH served as a loading control, and protein bands were quantified using ImageJ Software.

### 2.6. RNA Isolation and RT-qPCR

The analysis was conducted with the 2^-*ΔΔ*Cq^ quantification method as described by Livak and Schmittgen. Total RNA was isolated from cultured cells using a TRIzol® reagent (Invitrogen; Thermo Fisher Scientific Inc.) according to a previously published protocol. Briefly, 1 *μ*g total RNA was reverse-transcribed to cDNA using an AMV reverse transcriptase (Takara Biotechnology Co. Ltd., Dalian, China) and a RT primer. The reaction conditions were 16°C for 30 min, 42°C for 30 min, and 85°C for 5 min. Real-time PCR (RT-PCR) was performed using a TaqMan PCR kit on an ABI 7300 system (Applied Biosystems, USA) according to the manufacturer's protocol. The reactions were performed in a 96-well plate at 95°C for 10 min, followed by 40 cycles of 95°C for 10 sec and 60°C for 1 min. U6 was used as an internal control.

The relative expression levels of different mRNAs were using an AMV reverse transcriptase and oligo dT (Takara, Japan). The reaction conditions were 42°C for 60 min and 70°C for 10 min. Real-time PCR (RT-PCR) reactions were run on an ABI 7300 system (Applied Biosystems, USA) with SYBR Green dye and corresponding primers (Synthgene, Nanjing, China). The thermocycling conditions were as follows: 95°C for 5 min, followed by 40 cycles of 95°C for 30 sec, 60°C for 30 sec, and 72°C for 30 sec GAPDH was used as the endogenous control. Primer for c-Jun: forward primer: TGAGTGACCGCGACTTTTCA, reverse primer: TTTCTCTAAGAGCGCACGCA. Primer for GAPDH forward primer: 5′-GATATTGTTGACATCAATGAC-3′; GAPDH reverse primer: 5′-TTGATTTTGGAGGGATCTCG-3′. All primers are designed by primer5 software.

### 2.7. Plasmid Construction

The overexpression and empty plasmids used in this study were designed and synthesized by Synthgene (China). The overexpression plasmid sequence information of miR-214 is from the miRBase database, and the c-Jun overexpression plasmid sequence information is derived from the NCBI database. In the c-Jun knockdown experiment wherein we designed and synthesized siRNA, the sequence information is 5′-CAGUGUUGUUUGUAAAUAAGA-3′. Plasmids and siRNA were transfected into SCLC cells using Lipofectamine 2000 (Thermo Fisher Scientific, USA) according to the manufacturer's instructions.

### 2.8. Overexpression and Knockdown of miR-214

miR-214 overexpression and knockdown was achieved by transfecting cells with miR-214 mimics, negative control miRNA, anti-miR-214, and anti-miRNA negative control (Synthgene, China). Cells were seeded in 6-well plates and transfected using Lipofectamine 2000 (Thermo Fisher Scientific, USA). Total protein and total RNAs were extracted after the cells were transfected for 48 hrs and 24 hrs, respectively.

### 2.9. Migration Assay

The migratory abilities of the SCLC cells were investigated by using Transwell (24 well) chambers (Corning, USA). c-Jun was overexpressed or knocked down in SCLCs through transfection with c-Jun plasmid (1 *μ*g/well) or c-Jun siRNA (30 nM), respectively. The role of miR-214 on the migration of SCLCs was determined by transfection with nc (30 nM), miR-214 (30 nM), anti-miR-214 (30 nM), c-Jun plasmid (30 nM), or miR-214 (30 nM) plus c-Jun plasmid (1 *μ*g/well). The cells were then seeded into the upper Transwell chamber. Approximately 800 *μ*L of medium with 10% FBS was added to the lower chambers. Following incubation at 37°C and 5% CO_2_ for 24 h, the cells were fixed with 4% paraformaldehyde for 30 min and stained with crystal violet (Genview, China) for 20 min at room temperature. Cells on the upper side of the chamber were wiped off with a cotton bud. Cells from 3 random fields were captured and counted under a microscope.

### 2.10. Luciferase Reporter Assay

**Figure 1:**
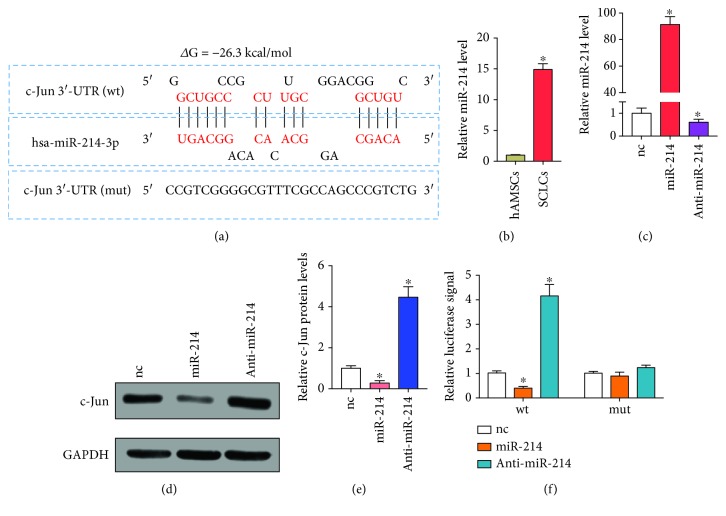
c-Jun is a direct target of miR-214. (a) Schematic illustration of the hypothetical duplexes formed through interactions between the binding site in the c-Jun 3′-UTR and miR-214. (b) RT-qPCR analysis of miR-214 levels in hAMSCs and SCLCs. (c) RT-qPCR analysis of miR-214 levels in SCLCs that were transfected with nc, miR-214, or anti-miR-214. (d, e) Western blotting analysis of c-Jun protein levels in SCLCs that were transfected with nc, miR-214, or anti-miR-214. GAPDH was used as the internal control: (d) representative image; (e) quantitative analysis. (f) Relative luciferase signals from SCLCs that were cotransfected with luciferase reporter gene (wt) or mutated luciferase reporter gene (mut) and nc, miR-214, or anti-miR-214. Data are shown as mean ± SEM (*n* = 3). ^∗^ *P* < 0.05, relative to nc.

The 3′-untranslated region (3′-UTR) sequence of c-Jun was derived from the NCBI database. The binding sequence of c-Jun that interacted with hsa-miR-214-3p was obtained by the BiBiServ2-RNAhybrid website (Figure 1(a)). The entire 3′-untranslated region (3′-UTR) of c-Jun containing the predicated target sites for hsa-miR-214-3p was synthesized by Synthgene (China). To investigate the binding specificity, the binding sequences that interacted with hsa-miR-214-3p were mutated (c-Jun wt 5′ GGCUGCCCCGCUUUGCGGACGGGCUGUC3′ c-Jun mut 5′ CCGTCGGGGCGTTTCGCCAGCCCGTCTG3′), and mutant c-Jun 3′-UTRs were then inserted into an equivalent luciferase reporter plasmid. In order to perform the luciferase reporter assay, the cells were plated in 24-well plates and 1 *μ*g luciferase reporter plasmid and 1 *μ*g *β*-galactosidase plasmid (internal control) were added to each well. After 4 h, the cell were transfected with 100 pmole of hsa-miR-214-3p mimics, negative control miRNA, anti-miR-214, anti-miRNA, and negative control using Lipofectamine® 2000 (Thermo Fisher Scientific Inc.), according to the manufacturer's protocol. The luciferase activity was measured using a Dual-Luciferase® Reporter Assay System in accordance with the manufacturer's protocol (Promega Corporation, Madison, WI, USA).

### 2.11. In Vivo Assay

After anesthetized SD rats were intraperitoneally injected with 2% pentobarbital sodium 50 mg/kg, the right sciatic nerve of rats was exposed under aseptic conditions and the sciatic nerve was excised 5 mm from the lower edge of the piriformis. The sciatic nerve was treated with medical suture. The grouping of animal experiments was as follows: A: sham; B: model; C: hAMSCs, after sciatic nerve injury modeling and injection of hAMSCs; D: SCLC group at the nerve injury, sciatic nerve injury modeling and injection of SCLCs; and E: in the SCLC+miR-214 group, SCLCs stably expressing miR-214 were injected into the nerve injury after modeling the sciatic nerve injury. 0.5 mL of type I collagen was prepared and mixed with hAMSCs or SCLCs (1 × 10^5^ cells/mL), and multipoint injection was applied to each wound (injection point is 1 mm from the midpoint of each side of the wound edge). In the control group, an equal amount of physiological saline was injected. SCLC cell lines stably expressing miR-214 were obtained by first constructing a miR-214 lentivirus, then a lentivirus infection of SCLCs, and finally obtaining stable expression cell lines by puromycin resistance screening (Synthgene, China).

The sciatic nerve function index was measured by the footprint method at 0, 2, 4, and 6 weeks postoperatively. The sciatic functional index (SFI) of each group was determined; specifically, after the ink was contaminated with the hind paw of the rat, the rat was let through the black box channel (8 cm∗8 cm) with white paper, the clear bilateral hind foot footprints of the rats were recorded, and the print length (PL) of the left and right feet and the distance from the first to fifth toes were measured (toe spread (TS), intermediate toe spread (TS)). E represents the experimental side and N represents the control side. SFI was calculated using the following formula:

(1) $$\begin{array}{c} \text{SFI}=\frac{109.5*\left(\text{ETS}-\text{NTS}\right)}{\text{NTS}}-\frac{38.3*\left(\text{EPL}-\text{NPL}\right)}{\text{NPL}}+\frac{13.3*\left(\text{EIT}-\text{NIT}\right)}{\text{NIT}}-8.8. \end{array}$$

### 2.12. Immunohistochemistry/Toluidine Blue Staining

The rats in each group of animals were subjected to isolation of the sciatic nerve tissue and embedded in paraffin to make paraffin sections. The MBP protein in the sample was stained by immunohistochemistry. The sections were incubated with a MBP monoclonal antibody (1 : 50 dilution) at 4°C overnight. The following day, samples were rinsed with PBS, incubated with biotin-conjugated secondary antibody at 37°C for 30 min, stained with diaminobenzidine for 30 sec, and counterstained with hematoxylin for 5 sec to visualize the nuclei. Images were taken under a microscope.

At the same time, the toluidine blue staining solution was used to stain the myelin tissue in the sample. The toluidine blue dye solution was diluted with water (the dilution with a 1 : 100 dilution). The slides were briefly soaked in the diluted staining solution, then soaked in water several times and fixed by tableting according to the selected method. Images were taken under a microscope.

### 2.13. Statistical Analysis

The results are expressed as the mean ± standard deviation of the mean of three independent experiments. Comparisons were determined using Student's *t*-test, and a *P* < 0.05 was considered statistically significant.

## 3. Results

### 3.1. Preparation of Schwann Cell-Like Cells

**Figure 2:**
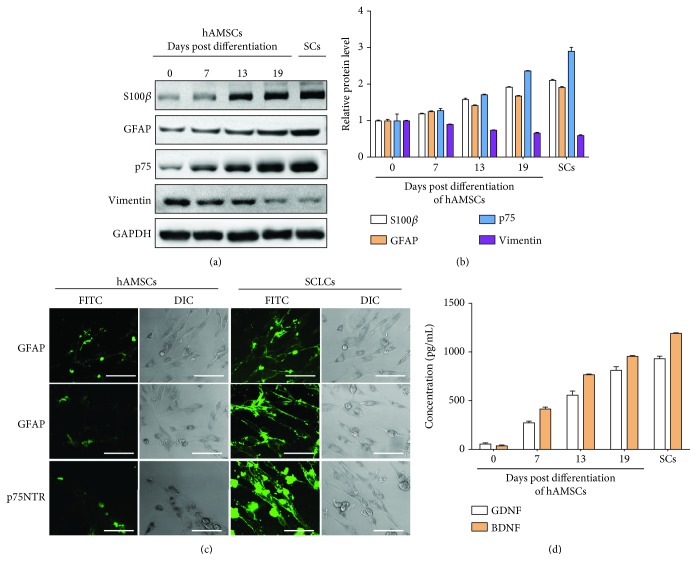
Validation of SCLCs that are derived from hAMSCs through differentiation. (a, b) Western blotting analysis of S100*β*, GFAP, and p75 protein levels in hAMSCs after differentiation in SC induction medium for 0, 7, 13, and 19 days and in SCs. GAPDH was used as the internal control: (a) representative image; (b) quantitative analysis. (c) Immunofluorescence staining of S100*β*, GFAP, and p75 in undifferentiated hAMSCs or SCLCs that were hAMSCs after 19 days' differentiation. Scale bar: 50 *μ*m. (d) Concentration of BDNF and GDNF in the culture supernatants of hAMSCs after differentiation in SC induction medium for 0, 7, 13, and 19 days and in the culture supernatants of SCs. Data are shown as mean ± SEM (*n* = 3).

We started the preparation of SCLCs by inducing differentiation of hAMSCs, which were firstly verified by flow cytometry analysis (Supplementary Figure 1). hAMSCs were cultured in SC induction medium to induce the transformation. To validate the formation of SCLCs, we analyzed the expression of SC-specific markers including S100*β*, glial fibrillary acidic protein (GFAP), and p75 [23, 36, 37] at 0, 7, 13, and 19 days after differentiation of hAMSCs. The protein levels of S100*β*, GFAP, and p75 were detected by western blotting assay. As shown in Figures 2(a) and 2(b), we observed gradual increases in the protein levels of S100*β*, GFAP, and p75, indicating that hAMSCs were successfully transformed into SCLCs. We also performed immunofluorescence staining of S100*β*, GFAP, and p75 in undifferentiated hAMSCs and SCLCs, further confirming the formation of SCLCs after 19 days' differentiation of hAMSCs (Figure 2(c)). Since SCs can secret neurotrophic factors such as brain-derived neurotrophic factor (BDNF) and glial cell-derived neurotrophic factor (GDNF) to promote PNR [18, 38], we also measured the expression levels of BDNF and GDNF in the culture supernatants of differentiated hAMSCs through enzyme-linked immunosorbent assay (ELISA). As shown in Figure 2(d), BDNF and GDNF levels in the culture supernatants kept increasing after differentiation of hAMSCs into SCLCs. To compare the behavior of SCLCs with that of SCs, we also checked the levels of SC-specific markers in SCs (Figures 2(a) and 2(b)) and expressions of secreted neurotrophic factors in the medium of cultured SCs (Figure 2(d)). Moreover, we measured the levels of vimentin [39] that is a MSC marker after the differentiation of hAMSCs (Figures 2(a) and 2(b)), confirming successful transformation of hAMSCs. All these results demonstrate that SCLCs can be obtained by inducing the differentiation of hAMSCs and SCLCs can also behave like SCs to secrete neurotrophic factors. Therefore, we used the SCLCs that were hAMSCs after differentiation for 19 days for the following studies.

### 3.2. Schwann Cell-Like Cells Promote the Functional Recovery of Sciatic Nerve Injury

**Figure 3:**
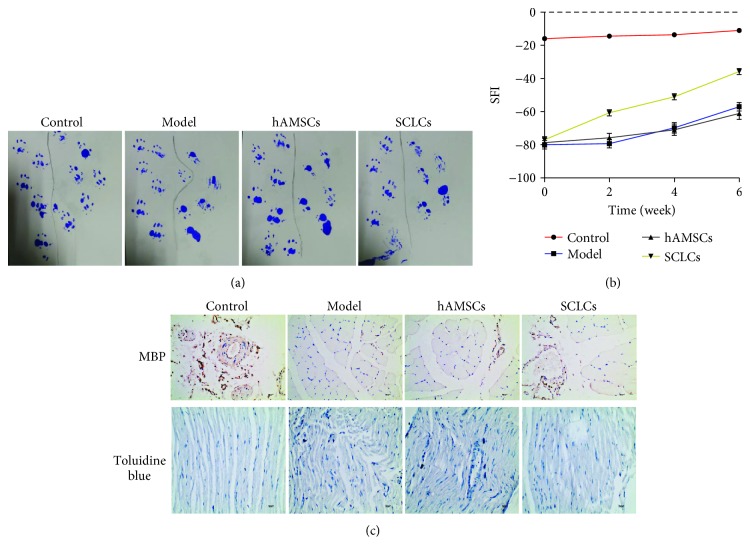
SCLCs promote PNR and functional recovery of sciatic nerve injury. Rats were divided into four groups, rats without treatment (control group), rats with sciatic nerve injury (model group), and rats with sciatic nerve injury and with transplantation of hAMSCs (hAMSC group) or SCLCs (SCLC group) into the injured site. (a) Representative footprint images. (b) SFI data that are quantitative analysis data of footprint results. (c) Immunohistochemical staining of dissected tissues. Data are shown as mean ± SEM (*n* = 5).

After successful preparation of SCLCs, we continued to investigate whether these SCLCs can promote PNR and functional recovery in a rat model of sciatic nerve injury. The rat models were developed by sciatic nerve transection at the right leg, followed by transplantation of hAMSCs or SCLCs into the injured sites. Rats were separated into four groups with 5 rats per group, including control groups, model groups with sciatic nerve injury, and hAMSC and SCLC groups that were composed of rats with sciatic nerve injury but transplanted with hAMSCs and SCLCs, respectively. We evaluated the functional recovery of sciatic nerve injury by using footprint assays. The representative footprint images after 6 weeks' recovery are shown in Figure 3(a). In the control groups, the paw footprints coordinated well. In the model groups, 6 weeks after injury, the footprints of the paws were remarkably mismatched. The introduction of hAMSCs into injured sites did not show improvements in the functional recovery. In contrast, SCLCs significantly promoted the functional recovery, even though it is still unsatisfactory. To quantify the footprint results, we recorded the footprint results of these four groups at 0, 2, 4, and 6 weeks after treatments and quantified them by calculating the sciatic function index (SFI). As shown in Figure 3(b), in the control groups, SFI values remained consistent and near to zero, suggesting that the function of the sciatic nerve is not affected. In the model groups, the SFI value was approximately -80 immediately following injury and increased slowly to ~57 after 6 weeks, confirming the loss of sciatic nerve function. Transplantation of hAMSCs into injured sites did not lead to significant improvements in the functional recovery. However, SCLCs after transplantation into injured sites significantly increased the SFI value from approximately 80 to approximately -36, validating that SCLCs can promote the functional recovery of sciatic nerve injury. Furthermore, we dissected these nerve tissues and stained them with the antibody for the myelination marker myelin basic protein (MBP) and toluidine blue (Figure 3(c)), confirming that SCLCs are able to facilitate sciatic nerve injury. Taken together, these results demonstrate that SCLCs have the ability to promote PNR and functional recovery of PNI.

### 3.3. c-Jun Is a Negative Regulator of the Myelination of Schwann Cell-Like Cells

Although stem cells have been extensively used to support PNR, the underlying mechanisms are still poorly understood. In our preliminary results, we found that SCLCs derived from hAMSCs showed therapeutic effect to improve PNR. However, the therapeutic outcome is still unsatisfactory. We envision that the understanding of molecular mechanisms involved in this process will provide hints to further enhance the therapeutic effect of SCLCs on PNR.

**Figure 4:**
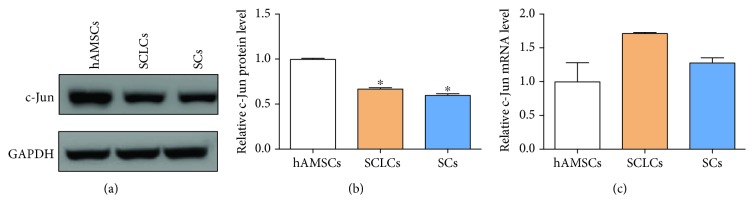
c-Jun is downregulated during the transformation of hAMSCs into SCLCs. (a, b) Western blotting analysis of c-Jun protein levels in hAMSCs, SCLCs, and SCs: (a) representative image; (b) quantitative analysis. (c) RT-qPCR analysis of c-Jun mRNA levels in hAMSCs, SCLCs, and SCs. Data are shown as mean ± SEM (*n* = 3). ^∗^ *P* < 0.05.

Since c-Jun is a key negative regulator of SC myelination [40], we first tried to study c-Jun in SCLCs that were derived from hAMSCs. We investigated the expression levels of c-Jun during the transformation of hAMSCs into SCLCs. As shown in Figures 4(a) and 4(b), the protein levels of c-Jun decreased after differentiation of hAMSCs into SCLCs, whereas the mRNA levels of c-Jun did not significantly change (Figure 4(c)). Since myelination is essential for SCs to promote PNR and c-Jun is downregulated during myelination [40], the downregulation of c-Jun protein levels in functional SCLCs suggests that c-Jun may also be a negative regulator of SCLC myelination. Moreover, the inconsistent mRNA and protein levels of c-Jun during the formation of SCLCs indicate that there is a posttranscriptional regulator in the upstream of c-Jun.

**Figure 5:**
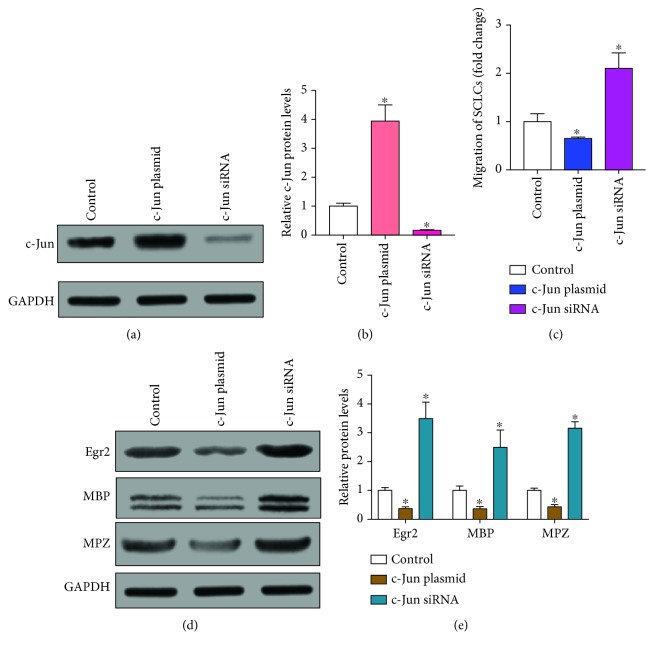
c-Jun negatively regulates the migration and myelination of SCLCs. c-Jun was overexpressed or knocked down in SCLCs through transfection with c-Jun plasmid or c-Jun siRNA, respectively. (a, b) Western blotting analysis of c-Jun protein levels in SCLCs that were transfected with c-Jun plasmid or c-Jun siRNA. GAPDH was used as the internal control: (a) representative image; (b) quantitative analysis. (c) Migration rates of SCLCs that were transfected with c-Jun plasmid or c-Jun siRNA. (d, e) Western blotting analysis of Egr2, MBP, and MPZ in SCLCs that were transfected with c-Jun plasmid or c-Jun siRNA: (d) representative image; (e) quantitative analysis. Data are shown as mean ± SEM (*n* = 3). ^∗^ *P* < 0.05, relative to control.

Next, we explored the role of c-Jun in SCLCs. We overexpressed and knocked down c-Jun in SCLCs. Overexpression of c-Jun was achieved through transfection of SCLCs with a plasmid that expresses the full open-reading frame of c-Jun. A siRNA that was designed to target the 3′-untranslated region (3′-UTR) of c-Jun was used to knock down c-Jun. The overexpression and knockdown of c-Jun was confirmed by analyzing protein levels of c-Jun in SCLCs (Figures 5(a) and 5(b)). We then checked the migration rates of SCLCs, showing that overexpression of c-Jun significantly inhibited the migration of SCLCs and downexpression of c-Jun enhanced the migration of SCLCs (Figures 5(c)). We also checked the expression levels of myelination markers in c-Jun-overexpressed or downregulated SCLCs, including MBP, zinc finger transcription factor Krox-20 (Egr2), and myelin protein zero (MPZ) [41, 42]. As shown in Figures 5(d) and 5(e), overexpression of c-Jun significantly downregulated the expression levels of Egr2, MBP, and MPZ, whereas inhibition of c-Jun upregulated their expression levels. Taken together, these results suggest that c-Jun is also a negative regulator of the migration and myelination of SCLCs.

### 3.4. c-Jun Is a Direct Target of miR-214

In light of the inconsistent mRNA and protein levels of c-Jun during the formation of SCLCs (Figure 4), we next continued to investigate the upstream posttranscriptional regulator of c-Jun. As miRNAs are typical posttranscriptional regulators [29], we used miRWalk to predict potential miRNAs that can directly regulate c-Jun. Among the identified candidate miRNAs, we selected miR-214 for further studies, since miR-214 can respond to sciatic nerve injury and is widely involved in the differentiation of stem cells [43–45]. Figure 1(a) shows the potential binding site of miR-214 in the 3′-UTR of c-Jun. The minimum free energy value of this hybridization was -26.3 kcal/mol. We also measured the expression levels of miR-214 by real-time quantitative PCR (RT-qPCR) during the differentiation of hAMSCs into SCLCs. As shown in Figure 1(b), a significant increase (~15-fold) in miR-214 levels was detected after the transformation of hAMSCs into SCLCs. These results suggest that miR-214 may be the upstream regulator of c-Jun in SCLCs.

To confirm the regulation between miR-214 and c-Jun in SCLCs, we overexpressed miR-214 in SCLCs through transfection with miR-214 mimics and downregulated miR-214 in SCLCs through transfection with antisense miR-214 (anti-miR-214) that blocks endogenous miR-214. The overexpression and inhibition of miR-214 in SCLCs were confirmed by RT-qPCR analysis, showing an ~91-fold increase in miR-214 levels after overexpression of miR-214 and an ~39% decrease in miR-214 levels after knockdown of miR-214 (Figure 1(c)). We then measured the protein levels of c-Jun in SCLCs, showing that overexpression of miR-214 significantly downregulated c-Jun protein levels while downregulation of miR-214 induced a significant increase in c-Jun protein levels (Figures 1(d) and 1(e)). To validate the negative regulation of miR-214 on c-Jun through the direct binding of miR-214 to the 3′-UTR of c-Jun (Figure 1(a)), we cloned the entire 3′-UTR of c-Jun into the 3′-UTR of luciferase. Through cotransfection of the miR-214 mimic or anti-miR-214 with the luciferase reporter gene into SCLCs, we could tell whether miR-214 directly binds to the 3′-UTR of c-Jun by measuring luciferase signals. As shown in Figure 1(f), a significant decrease in luciferase signals (~60%) was induced by miR-214, whereas anti-miR-214 caused a significant increase in luciferase signals (~4.1-fold). We also mutated the 3′-UTR of c-Jun and inserted it again into the 3′-UTR of luciferase. When using the mutated luciferase reporter gene to perform the same experiments, we did not find significant changes in luciferase signals after overexpression or inhibition of miR-214 in SCLCs. Taken together, these results demonstrate that miR-214 directly targets c-Jun to repress its expression.

### 3.5. miR-214 Targets c-Jun to Promote the Myelination of Schwann Cell-Like Cells

**Figure 6:**
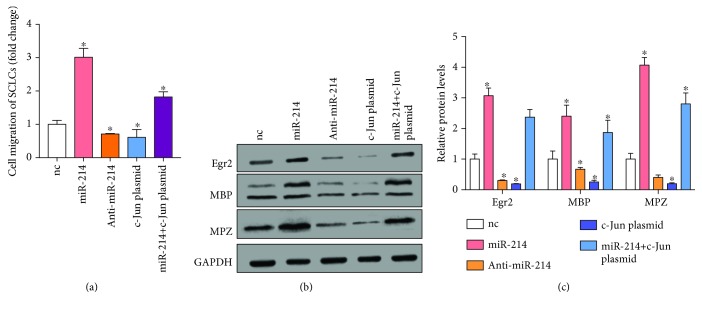
miR-214 promotes the migration and myelination of SCLCs. (a) Migration rates of SCLCs that were transfected with nc, miR-214, anti-miR-214, c-Jun plasmid, or miR-214 plus c-Jun plasmid. (b, c) Western blotting analysis of Egr2, MBP, and MBZ protein levels in SCLCs that were transfected with nc, miR-214, anti-miR-214, c-Jun plasmid, or miR-214 plus c-Jun plasmid. GAPDH was used as the internal control: (b) representative image; (c) quantitative analysis. Data are shown as mean ± SEM (*n* = 3). ^∗^ *P* < 0.05, relative to nc.

As c-Jun is a negative regulator of the myelination of SCLCs and is regulated by miR-214, we hypothesized that overexpression of miR-214 could further enhance SCLC myelination through downregulation of c-Jun. To validate this, we overexpressed or knocked down miR-214 in SCLCs. We also simultaneously overexpressed miR-214 and c-Jun in SCLCs to investigate whether the effect of miR-214 on SCLCs was through targeting c-Jun. We then measured the migration rates of these transfected SCLCs (Figure 6(a)), showing that overexpression of miR-214 promoted the migration of SCLCs (~3-fold) while downregulation of miR-214 inhibited the migration of SCLCs (~29%). It is noteworthy that concurrent overexpression of miR-214 and c-Jun alleviated the positive effect of miR-214 on the migration of SCLCs. We also measured the expression levels of myelination markers Egr2, MBP, and MPZ (Figures 6(b) and 6(c)), showing that miR-214 significantly upregulated the expression of myelination makers and concurrent overexpression of c-Jun prevented the increase of myelination markers in SCLCs. These results indicate that miR-214 promotes the migration and myelination of SCLCs through targeting c-Jun.

### 3.6. miR-214 Overexpressed in Schwann Cell-Like Cells Enhances the Functional Recovery of Sciatic Nerve Injury

**Figure 7:**
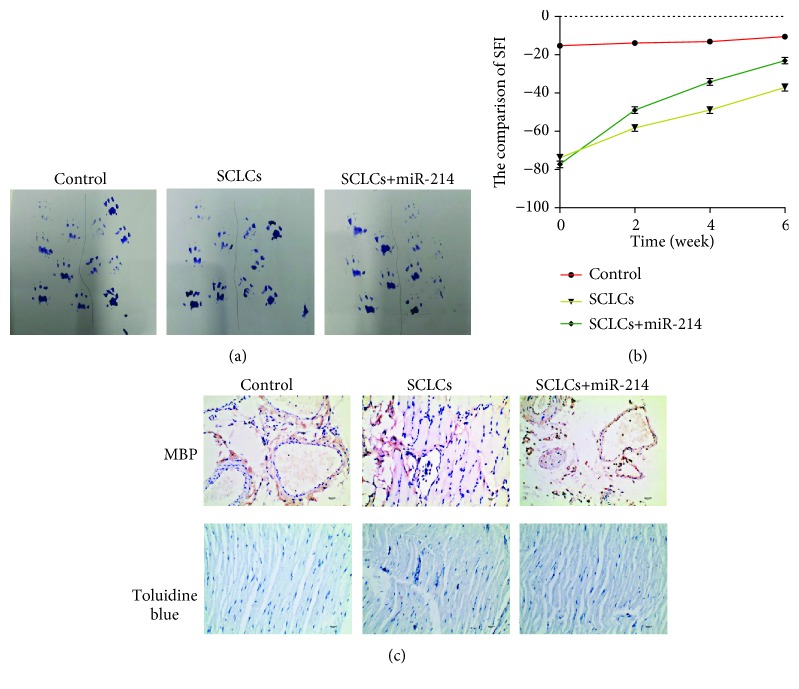
miR-214 upon overexpression in SCLCs enhances the therapeutic outcomes of SCLCs in PNR and the functional recovery of sciatic nerve injury. Rats were divided into three groups, rats without treatment (control group), rats with sciatic nerve injury and with transplantation of SCLCs (SCLC group) or miR-214-overexpressed SCLCs into the injured site. (a) Representative footprint images. (b) SFI data that are quantitative analysis data of the footprint results. (c) Immunohistochemical staining of dissected tissues. Data are shown as mean ± SEM (*n* = 5).

Finally, to investigate whether overexpression of miR-214 in SCLCs could further promote PNR after transplantation of SCLCs into the injured sites, we first established SCLCs stably expressing miR-214 by using a lentivirus vector. We then developed rat models of sciatic nerve injury, followed by transplantation of SCLCs or miR-214-overexpressed SCLCs into the injured sites. Footprints were recorded and SFI values were quantified by using the same method as described above. As shown in Figure 7(a), in comparison with that in the SCLC group, a significant improvement in the coordination of paw footprints in miR-214-overexpressed SCLC groups was observed. We also detected a remarkable improvement in SFI values in miR-214-overexpressed SCLC groups, which were from ~80 to ~25 (Figure 7(b)). Immunohistochemical staining of dissected tissues further confirmed these results (Figure 7(c)). Overall, we demonstrate that overexpression of miR-214 in SCLCs could further promote PNR and functional recovery of sciatic nerve injury.

## 4. Discussion and Conclusion

PNI is a common problem worldwide, causing long-term morbidity and disability as well as economic burden. It has been estimated that the incidence of PNI in developed countries is approximately 18/100,000 every year, whereas it is even higher in developing countries [46, 47]. There are many factors that can lead to PNI, including trauma, surgery, cancer, and anesthesia injections [7]. Despite great improvements in microsurgical techniques, efficient therapeutic strategies for PNI are still lacking and are in high demand. Many efforts have been devoted to the development of therapeutic strategies for PNR [42, 48, 49]. SCs that are glia in PNS were considered as promising agents to promote PNR and functional recovery. However, several limitations as discussed above still hinder the clinical translation of SCs in PNR. Regenerative medicine using stem cells that can transform into SC precursor cells or SCLCs has displayed great promise in the treatment of PNI. Here, we used hAMSCs as the cell sources to produce SCLCs after induction with a SC differentiation medium. Through measuring specific markers of SCs, we confirmed the formation of SCLCs (Figure 2). Moreover, after transplantation of SCLCs into the injured sites in a rat model of sciatic nerve injury, we validated that SCLCs derived from hAMSCs could promote PNR and the functional recovery of sciatic nerve injury (Figure 3).

Although there is a great enthusiasm for exploiting stem cells to generate SCLCs for PNR [11, 28, 50], the underlying mechanisms for this therapeutic outcome are still poorly understood. Meanwhile, whether these stem cells can cause side effects is still unknown and the therapeutic results of using stem cells in PNI are also still unsatisfactory. Understanding the molecular mechanisms will not only provide molecular basis for using the stem cells to treat PNI but also afford a practical method to enhance the stem cell-based therapy in PNR. In our studies, we started investigation of the molecular mechanism underlying SCLC-promoted PNR with a negative regulator of SC myelination, which is c-Jun [40]. Although c-Jun is previously identified as a positive regulator of migration in solid tumors [51–53], here we found that c-Jun negatively regulated the migration and myelination of SCLCs (Figure 5). These results were consistent with the previous report regarding the role of c-Jun in SC myelination [40]. Moreover, we observed inconsistent expression levels of c-Jun mRNA and protein during the transformation of hAMSCs into SCLCs (Figure 4). This result indicates that there exists a posttranscriptional regulator in the upstream of c-Jun, which was identified as miR-214 (Figure 1). Based on this novel pathway, we further demonstrated that miR-214 had a positive impact on PNR through targeting c-Jun and overexpression of miR-214 in SCLCs could further improve the therapeutic effect of SCLCs in PNR (Figures 6 and 7). Collectively, these results suggest that SCLCs derived from hAMSCs promote PNR through a miR-214/c-Jun pathway and miR-214 is an ideal target for future therapeutic treatment of PNI through using SCLCs.

On the other hand, we have only revealed one endogenous pathway inside SCLCs that mediated the therapeutic effects of SCLCs. How to further unveil the molecular mechanisms in details may further provide hints for treatment of PNI. Exosomes are a class of extracellular vesicles that contain numerous functional biomolecules and mediate cell-to-cell communication [54]. Recent results have indicated that stem cells or SCs can secret exosomes and these exosomes can be used independently to promote PNR [55, 56]. However, the detailed molecular mechanisms about which content in exosomes promotes PNR are still unknown. It is noteworthy that previous reports have also revealed that exosomes can transport miRNAs between different cells [57]. Therefore, in our studies, whether SCLCs derived from hAMSCs can secret exosomes that contain miR-214 to promote PNR is worth exploring and is now under investigation in our group.

In conclusion, we prepared SCLCs by inducing the differentiation of hAMSCs and validated SCLCs by detecting SC markers. SCLCs showed great potential in promoting the repair of sciatic nerve injury. We also unveiled a novel pathway in SCLCs that mediated their therapeutic effect, which is when miR-214 targets c-Jun to promote the therapeutic effect of SCLCs in PNR. Moreover, SCLCs overexpressed with miR-214 showed enhanced effect in the repair of PNI. Overall, our results prove that SCLCs have potential beneficial effect for myelination. Thus, our results provide a novel strategy for not only obtaining seed cells for use in nerve engineering but also enhancing the therapeutic outcomes of these seed cells. More detailed molecular mechanisms underlying this process and the combined use of SCLCs derived from hAMSCs and exosomes with high expression of miR-214 in PNR are now underway in our group.

## Data Availability

The data used to support the findings of this study are included within the article.

## Supplementary Materials

Supplementary Materials Supplementary Figure 1 Flow cytometry analysis of CD34, CD45, CD29, and CD44 markers in hAMSCs.
