# Supplementary figures and images for: Schwann Cell-Like Cells Derived from Human Amniotic Mesenchymal Stem Cells Promote Peripheral Nerve Regeneration through a MicroRNA-214/c-Jun Pathway
Source: Stem Cells Int. 2019 Jul 1;2019:2490761. doi: 10.1155/2019/2490761 (PMC6636479; doi:10.1155/2019/2490761)

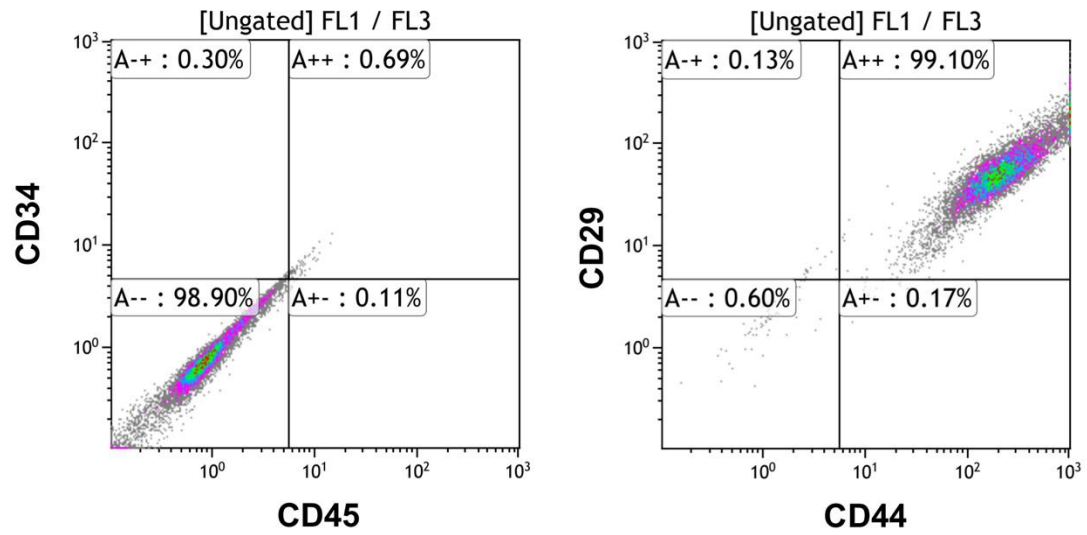

Supplementary Figure 1. Flow cytometry analysis of CD34, CD45, CD29 and CD44 markers in hAMSCs.

Supplement: Supplementary Materials — Supplementary Figure 1 Flow cytometry analysis of CD34, CD45, CD29, and CD44 markers in hAMSCs. [file 2490761.f1.pdf]
